# Exploring the Mechanism of Shexiang Baoxin Pill in the Treatment of Ischemic Stroke: A Study Integrating Network Pharmacology, Machine Learning, Molecular Docking, and Molecular Dynamics Simulation

**DOI:** 10.1155/bmri/9932683

**Published:** 2026-06-03

**Authors:** Jingju Fu, Yukun Wang, Xinyi Li, Xue Dong

**Affiliations:** ^1^ Institute of Traditional Chinese Medicine Chemistry, Shandong Academy of Chinese Medicine, Jinan, Shandong, China, sdfmu.edu.cn

**Keywords:** bioinformatics, ischemic stroke, machine learning, molecular docking, molecular dynamics simulation, Shexiang Baoxin Pill

## Abstract

Ischemic stroke (IS) is a major global cause of mortality and long‐term disability and is closely associated with cardiovascular disease (CVD). Its pathogenesis overlaps with cardiovascular disorders, particularly atherosclerosis and immune–inflammatory dysregulation. Shexiang Baoxin Pill (SBP), a traditional Chinese medicine used for coronary heart disease and angina pectoris, has been reported to improve microcirculation and alleviate inflammation; however, the mechanisms of SBP against IS remain unclear. Here, an integrated strategy combining network pharmacology, machine learning, molecular docking, and molecular dynamics simulation was applied to explore the potential mechanisms of SBP in IS. In total, 126 bioactive compounds and 796 potential targets of SBP were identified. Intersection analysis with IS‐related differentially expressed genes from GEO datasets identified six candidate targets: CXCL8, IL1B, JUN, NR4A2, PTGS2, and TNF. Functional enrichment analysis suggested that these targets were mainly involved in cardiovascular and cerebrovascular processes, including inflammatory responses, immune regulation, and apoptosis. Combined protein–protein interaction network analysis and machine learning further identified TNF and JUN as hub genes related to IS. Molecular docking and molecular dynamics simulations suggested stable binding between representative active ingredients (e.g., bufalin and bufotalin) and the two hub targets, and the TNF‐compound complexes remained structurally stable during the simulation. Collectively, this computational study suggests that SBP may exert therapeutic effects in IS through a multicomponent and multitarget regulatory network involving inflammation‐ and immunity‐related pathways. These findings provide preliminary molecular evidence and a basis for further experimental validation of the cerebrovascular protective effects of SBP.

**Highlights**

• An integrative framework combining network pharmacology, machine learning, and molecular dynamics explores potential mechanisms of Shexiang Baoxin Pill in ischemic stroke.
• JUN and TNF are identified as key inflammatory hub genes potentially involved in the cardiovascular–cerebrovascular pathological network.
• Core bioactive components of SBP exhibit stable binding to pivotal targets, supporting its multicomponent and multitarget pharmacological characteristics.

## 1. Introduction

Cerebrovascular diseases, particularly ischemic stroke (IS), are a major global health burden and rank among the leading causes of death and long‐term disability [1]. IS accounts for over 70% of stroke cases, causing millions of fatalities annually and imposing substantial socioeconomic costs, exacerbated by aging populations and rising cardiovascular risk factors [2, 3]. The pathogenesis of IS is closely linked to systemic cardiovascular disorders, including atherosclerosis and endothelial dysfunction, highlighting a shared heart–brain vascular continuum [4]. Acute artery occlusion triggers ischemia‐driven cascades of energy failure, excitotoxicity, oxidative stress, and inflammation, culminating in irreversible neuronal injury [5].

Current therapeutic strategies, such as thrombolysis and thrombectomy, are limited by narrow time windows, eligibility criteria, and hemorrhagic risk [6, 7]. Conventional neuroprotective or preventive approaches mainly target vascular risk factors, providing limited direct benefit for postischemic neuronal recovery [8–11]. These limitations underscore the need for novel, multitarget strategies capable of modulating the complex pathological cascades in IS [12].

In this context, traditional Chinese medicine (TCM), characterized by multicomponent and multitarget regulatory properties, is increasingly recognized as a complementary approach for complex cardiovascular and cerebrovascular diseases [13, 14]. Botanical drugs aimed at “invigorating blood and transforming stasis” dominate TCM prescriptions for these conditions, functioning to restore vascular patency and enhance microcirculation, thereby exerting therapeutic effects against ischemic pathologies [15]. Shexiang Baoxin Pill (SBP), a Chinese patent medicine derived from classical formulations, has been widely applied in cardiovascular diseases such as coronary heart disease and angina [16, 17].

Based on an analysis of pharmacological monographs and standards, the utilization frequency of 398 Chinese herbal medicines for IS treatment was evaluated. *Moschus* (Shexiang) ranked fourth and was identified as a primary agent for promoting blood circulation and restoring vascular patency, whereas other key herbs, including *Borneolum Syntheticum* (Bingpian), *Bovis Calculus* (Niuhuang), *Cinnamomi Cortex* (Rougui), and *Ginseng Radix et Rhizoma* (Renshen), were among the top 50 most commonly used ingredients [18]. The high degree of compositional overlap between these frequently used herbs and the core constituents of SBP suggests a potential framework for shared therapeutic strategies in cardio‐cerebrovascular diseases, highlighting the intrinsic link within the brain–heart axis and their analogous pathogenic mechanisms.

Supporting this concept, preclinical studies have indicated that Shexiang Tongxin Dropping Pill, a Chinese patent medicine primarily prescribed for angina pectoris, can restore cerebral microvascular function in animal models [19]. Specifically, SBP has been reported to ameliorate cognitive and locomotor deficits in IS models, potentially via inhibition of neuronal apoptosis and promotion of hippocampal synaptic neuroplasticity [20]. Collectively, these findings provide both pharmacological and experimental evidence for the feasibility of “simultaneous treatment of the brain and heart” through unified multicomponent interventions.

**Figure 1:**
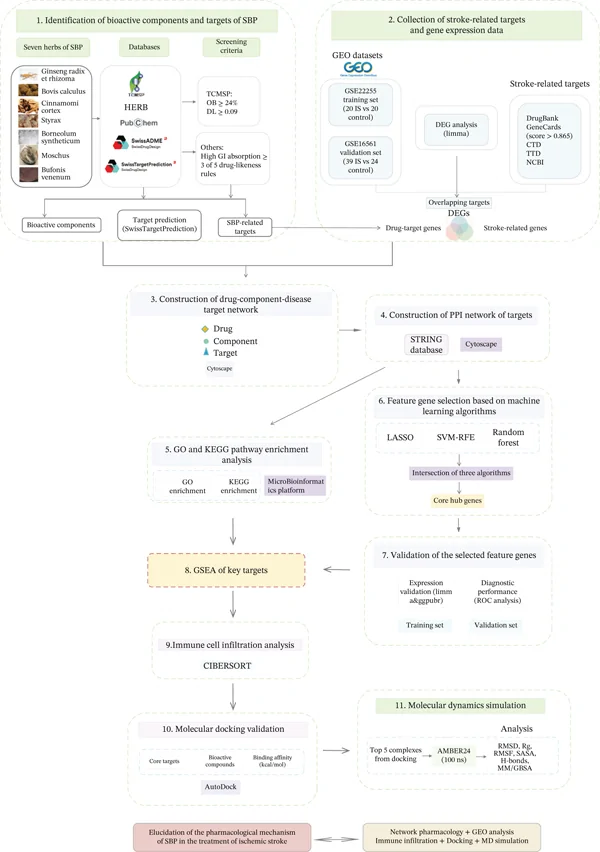
Schematic overview of the study workflow. The schematic diagram shows the integrated research strategy used to investigate the pharmacological mechanism of SBP in treating IS, from target screening to multilevel validation.

Nevertheless, the precise molecular targets, signaling networks, and system‐level mechanisms through which SBP exerts its effects in IS remain largely undefined, limiting mechanistic understanding and evidence‐based clinical application. Advances in computational pharmacology and artificial intelligence provide powerful tools to deconvolute complex multicomponent formulations [21]. Accordingly, this study employs an integrated in silico framework—including network pharmacology, machine learning, molecular docking, and molecular dynamics (MD) simulations—to systematically investigate SBP mechanisms in IS. Bioactive compounds and putative targets were identified from TCM databases and intersected with IS‐related genes and differentially expressed genes (DEGs) from GEO datasets [22]. Functional enrichment, protein–protein interaction (PPI) networks, and machine learning feature selection were subsequently used to identify key hub targets and bioactive components. Molecular docking and dynamics simulations further assessed the stability of compound–target interactions. This approach provides a systematic, multitarget perspective of SBP against IS, offering preliminary theoretical support for the “simultaneous treatment of brain and heart” strategy. Before detailing the methodology and results, we first present the overall workflow in Figure 1.

## 2. Materials and Methods

### 2.1. Identification of Bioactive Components and Targets of SBP

Bioactive components of the five constituent herbs of SBP—*Ginseng Radix et Rhizoma*, *Bovis Calculus*, *Cinnamomi Cortex*, *Styrax*, and *Borneolum Syntheticum*—were identified from the TCMSP (https://tcmsp-e.com/tcmsp.php) using oral bioavailability (OB ≥ 24%) and drug‐likeness (DL ≥ 0.09) as selection criteria. For compounds from *Moschus* and *Bufonis Venenum* not recorded in TCMSP, additional databases, including HERB (http://herb.ac.cn), PubChem (https://pubchem.ncbi.nlm.nih.gov/), SwissADME (https://www.swissadme.ch/), and SwissTargetPrediction (https://www.swisstargetprediction.ch/), were integrated for compound identification; eligible compounds were required to exhibit high gastrointestinal (GI) absorption and to meet at least three of five DL rules (Lipinski, Ghose, Veber, Egan, and Muegge). The canonical SMILES of all selected compounds were then submitted to SwissTargetPrediction with the target species set to Homo sapiens, and potential targets were predicted using a threshold of Probability > 0, with the resulting compound–target interactions collected for subsequent analysis.

### 2.2. Collection of Stroke‐Related Targets and Gene Expression Data

Gene expression profiles and raw CEL files from the GSE22255 dataset, generated using the Affymetrix Human Genome U133A platform, were downloaded from the Gene Expression Omnibus (GEO) database (https://www.ncbi.nlm.nih.gov/geo/) and used as the training cohort; this dataset included 40 samples, comprising 20 healthy controls and 20 IS samples. The GSE16561 dataset from GEO, containing 63 samples (24 healthy controls and 39 IS samples), was selected as an independent validation cohort for subsequent verification of candidate gene markers. Probe identifiers were mapped to corresponding gene symbols, and DEGs between IS and control samples were identified using the limma package in R, with thresholds of |log2 fold change (log2FC)| > 1.5 and a *p* value < 0.05; volcano plots were generated to visualize DEG distribution and expression patterns. Stroke‐related disease targets were systematically retrieved from the DrugBank (https://go.drugbank.com/), GeneCards (https://www.genecards.org/), Comparative Toxicogenomics Database (CTD, https://ctdbase.org/), Therapeutic Target Database (TTD, https://ttd.idrblab.cn/), and NCBI databases (https://www.ncbi.nlm.nih.gov/) using the keywords “stroke,” “ischemic stroke,” and “hemorrhagic stroke,” with GeneCards targets subsequently filtered by a relevance score > 0.865 to increase specificity. Finally, overlapping genes between DEGs and stroke‐related targets were identified using MicroBioinformatics Platform (https://www.bioinformatics.com.cn/), and the shared targets were visualized using Venn diagrams.

### 2.3. Construction of the Drug–Component–Disease Target Network

A multilevel interaction network of potential therapeutic targets for IS was constructed using Cytoscape software (Version 3.10.1) to visualize the relationships among SBP, its bioactive components, and disease‐related targets. In the network, nodes represented the drug, compounds, or targets, whereas edges denoted the interactions among them. Topological parameters of all nodes were systematically evaluated using the Network Analyzer tool and the cytoNCA plugin, enabling quantitative assessment of the structural and functional importance of each node within the network.

### 2.4. Construction of the PPI Network

The potential therapeutic targets identified above were imported into the STRING database (https://www.string-db.org/), with the organism restricted to Homo sapiens. To construct the PPI network, a minimum required interaction score of 0.4 was applied, and isolated nodes were removed. The resulting network was visualized and preliminarily analyzed using Cytoscape software (Version 3.10.1). Subsequently, topological properties of the PPI network were systematically evaluated using the Network Analyzer tool and the cytoNCA plugin to identify key nodes based on network topology parameters.

### 2.5. GO and KEGG Pathway Enrichment Analysis

The identified potential key targets of SBP in the treatment of IS were uploaded to the MicroBioinformatics Platform (https://www.bioinformatics.com.cn/) for Gene Ontology (GO) functional annotation and Kyoto Encyclopedia of Genes and Genomes (KEGG) pathway enrichment analysis. During the analysis, the species was set to Homo sapiens, and a *p* value < 0.05 was considered statistically significant. Based on the number of enriched target genes, the most significantly enriched biological processes and signaling pathways were selected for further interpretation.

### 2.6. Feature Gene Selection Based on Machine Learning Algorithms

To identify IS‐associated core genes, three machine learning algorithms—least absolute shrinkage and selection operator (LASSO) regression, support vector machine–recursive feature elimination (SVM‐RFE), and random forest (RF)—were jointly applied using the R packages glmnet, e1071, and randomForest, respectively. LASSO regression employs L1 regularization to shrink regression coefficients toward zero, thereby eliminating redundant variables and enhancing model sparsity and generalizability [23], whereas SVM‐RFE iteratively removes features with minimal contribution to classification performance to refine the optimal gene subset [24]. The RF algorithm aggregates multiple decision trees and ranks features according to their importance in classification accuracy, making it well suited for high‐dimensional and noisy biological data [25]. To ensure robustness and reliability, only genes consistently identified by all three algorithms were retained and defined as IS‐specific core genes.

### 2.7. Validation of the Selected Feature Genes

To evaluate the diagnostic performance of IS‐specific core genes, differential expression analysis was first performed in the training cohort using the limma package, and the ggpubr package was applied to visualize expression differences between IS samples and healthy controls. Receiver operating characteristic (ROC) curves were then generated using the pROC package, and the area under the curve (AUC) was calculated to quantify the discriminative ability of each core gene [26]. To further evaluate the robustness of the model, an independent external validation cohort was analyzed, in which the expression patterns of the core genes were examined and ROC analyses were repeated to assess their diagnostic performance across datasets.

### 2.8. Immune Cell Infiltration Analysis

To investigate the immune cell composition within the samples, the CIBERSORT algorithm (https://cibersort.stanford.edu/) was applied to estimate the relative abundance of immune cell populations in the training datasets [27]. This approach uses linear support vector regression to deconvolute gene expression matrices and infer the proportions of distinct immune cell subtypes. Immune cell infiltration analysis was performed using R software, and samples meeting the filtering criterion of *p* < 0.05 were retained to generate the immune cell expression matrix for subsequent analyses.

### 2.9. Molecular Docking Validation

Molecular docking analyses were performed to predict the interactions between the identified bioactive compounds of SBP and the core targets (TNF and JUN). The crystal structures of the target proteins were obtained from the RCSB Protein Data Bank (PDB) (TNF, PDB ID: 5UUI; JUN, PDB ID: 5 T01), and the molecular structures of bioactive compounds were retrieved from the PubChem database. Protein and ligand structures were preprocessed using PyMOL software, including removal of water molecules, addition of polar hydrogen atoms, and assignment of Gasteiger charges. The prepared structures were saved in the PDBQT format for docking.

Docking simulations were performed using AutoDock, with grid boxes defined to fully encompass the predicted binding regions of each target protein. For JUN, the grid box (center: −23.326, 17.31, 20.956 Å; size: 100.375 × 100.375 × 100.375 Å; 126 × 126 × 126 grid points; spacing = 0.803 Å) covered the entire leucine zipper interface, which lacks a canonical small‐molecule binding pocket. For TNF, the grid box (center: 45.3, 52.929, 13.77 Å; size: 101 × 101 × 101 Å; 126 × 126 × 126 grid points; spacing = 0.808 Å) encompassed the homotrimer interface. Relatively large grid boxes and identical parameters across all ligands were applied to ensure full coverage of potential functional and allosteric sites and to maintain comparability of predicted binding affinities. Other AutoDock parameters were set according to recommended defaults to ensure reproducibility.

The docking protocol was validated to ensure reliability. For TNF, which contains a cocrystallized ligand, the ligand was extracted and redocked into the defined binding pocket using the same docking parameters and scoring function. The root‐mean‐square deviation (RMSD) between the redocked conformation and the original crystallographic ligand was calculated. An RMSD < 2.0 Å suggested that the docking parameters and grid setup could reliably reproduce the experimentally determined binding mode. Since JUN lacks a cocrystallized ligand, conventional redocking validation could not be performed. To allow relative comparison of predicted binding affinities across ligands, the docking parameters previously validated for TNF were applied to a large grid encompassing the leucine zipper interface of JUN. It should be noted that these results are predictive and intended solely for ranking purposes, and do not constitute experimental validation of absolute binding modes. Docking scores were interpreted using standardized binding affinity thresholds: ≤ −7.0 kcal/mol as strong binding, −5.0 to −7.0 kcal/mol as moderate binding, and ≥ −5.0 kcal/mol as weak binding. Only compounds with moderate or strong binding affinities were considered potentially effective. The predicted interactions between bioactive compounds and target proteins were visualized and analyzed using PyMOL software [28].

### 2.10. MD Simulation

MD simulations were performed using the AMBER24 software package to investigate the dynamic behavior of the five protein–ligand complexes exhibiting the highest binding affinities in the preliminary identification. Each system was simulated for a total duration of 100 ns [29]. The AMBER19SB force field was applied for proteins, whereas the GAFF2 force field was used for ligands. An explicit solvent environment was constructed using the TIP3P water model. Prior to simulation, each system underwent energy minimization using the steepest descent algorithm to eliminate unfavorable contacts and relieve local structural strain. Subsequently, system equilibration was carried out in two stages. First, the system was gradually heated from 0 to 300 K under the canonical ensemble (NVT) with a time step of 1 fs over 100 ps, during which weak positional restraints of 15 kcal/(mol Å) were applied to the protein backbone atoms. This was followed by equilibration under the isothermal–isobaric ensemble (NPT), maintaining the pressure at 1.0 bar to ensure proper system density and thermodynamic stability.

Production MD simulations were conducted with an integration time step of 2 fs, and trajectory coordinates were recorded every 100 ps for subsequent analysis. To evaluate the dynamic stability and conformational behavior of the protein structures, several trajectory‐based parameters were calculated. Root mean square deviation (RMSD) was used to assess conformational drift of the protein backbone relative to the initial structure [30]. The radius of gyration (Rg) was calculated to reflect the compactness of the overall protein structure [31]. Root mean square fluctuation (RMSF) was employed to characterize residue‐level flexibility and local dynamic fluctuations [32]. Solvent‐accessible surface area (SASA) was computed to describe the extent of solvent exposure of the protein surface, providing insight into hydrophobic core stability and conformational changes. In addition, the number of hydrogen bonds (H‐bonds) was analyzed to evaluate hydrogen‐bonding networks within the protein and between the protein and solvent, thereby identifying key interactions contributing to structural stability. Furthermore, binding free energy calculations were performed using the molecular mechanics/generalized Born surface area (MM/GBSA) method, implemented through the MMPBSA.py module in the AMBER24 package, to quantitatively estimate the binding affinities between proteins and ligands.

## 3. Results

### 3.1. Identification of Active Components and Targets of SBP

Based on the TCMSP database, active ingredients from *Ginseng Radix et Rhizoma*, *Bovis Calculus*, *Cinnamomi Cortex*, *Styrax*, and *Borneolum Syntheticum* contained in SBP were systematically retrieved and screened following preset inclusion criteria. Meanwhile, active components of *Moschus* and *Bufonis Venenum* were further supplemented from the HERB, PubChem, SwissADME, and SwissTargetPrediction databases and filtered using the same standardized workflow.

In total, 126 active components were retrieved for SBP (Table S1), which corresponded to 796 nonredundant candidate targets after deduplication (Table S2). Table S1 lists the detailed information of these compounds, and Table S2 summarizes their predicted potential targets. These components were distributed among seven herbal materials: *Moschus* (19), *Ginseng Radix et Rhizoma* (47), *Bovis Calculus* (8), *Cinnamomi Cortex* (13), *Styrax* (13), *Bufonis Venenum* (21), and *Borneolum Syntheticum* (5). This observed multicomponent and multitarget distribution pattern is consistent with the typical holistic characteristics of TCM formulations.

### 3.2. Identification of IS‐Related Targets and DEGs

**Figure 2:**
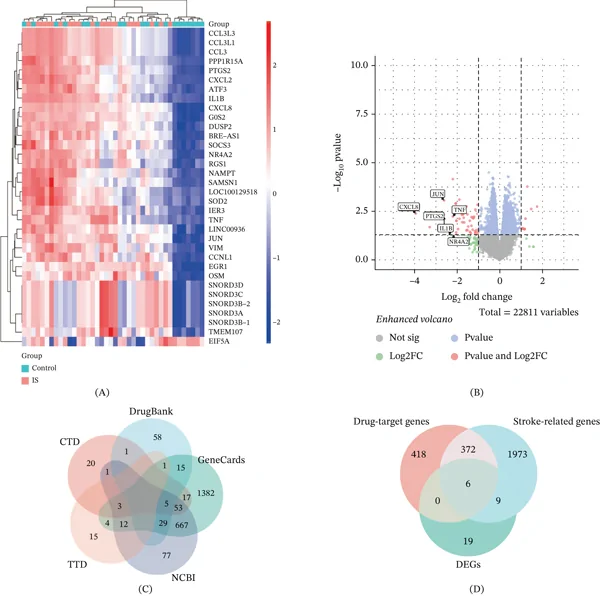
Differential gene expression analysis between the IS and control groups and multisource integrative intersection analysis of IS‐related genes. (A) Heatmap illustrating the top 34 DEGs, comprising the one most strongly upregulated and 33 most downregulated transcripts. Expression intensity is visualized using a color scale, where warmer tones denote elevated expression and cooler tones correspond to lower levels. Hierarchical clustering was applied to both gene and sample axes to reveal grouping patterns. (B) Volcano plot depicting the genome‐wide expression profile. Significantly upregulated and downregulated genes are highlighted in distinct colors, whereas nonsignificant genes are shown in neutral tones. Genes with the highest statistical significance (|log2FC| > 1.5, *p* < 0.05) are specifically annotated. (C) Venn diagram displaying the unified gene set compiled from several public resources, including GeneCards, CTD, DrugBank, TTD, and NCBI. After removing redundant entries, 2360 unique genes associated with IS were retained. (D) Venn diagram representing the overlap among IS‐related genes, GEO‐derived DEGs, and drug–target genes.

Gene expression profiles were analyzed via the GEO2R tool in the GEO database. DEGs were screened under the thresholds of *p* < 0.05 and |log2FC| > 1.5, with detailed information provided in Table S3. The expression patterns of these DEGs were visualized by a heat map (Figure 2A) and volcano plot (Figure 2B), which displayed apparent transcriptional differences between IS and control samples.

A total of 34 IS‐associated DEGs were obtained, including 33 downregulated and one upregulated gene. Meanwhile, 2360 IS‐related genes were collected from the DrugBank, GeneCards, CTD, TTD, and NCBI databases, as shown in Figure 2C. These disease‐related genes were further intersected with the 34 GEO‐derived DEGs and 796 SBP‐related candidate targets, and the overlapping results are presented in Figure 2D.

Based on this integrative screening strategy, six intersecting genes, namely CXCL8, IL1B, JUN, NR4A2, PTGS2, and TNF, were finally obtained. These genes may be potentially correlated with IS pathogenesis and could serve as candidate therapeutic targets related to SBP.

### 3.3. Compound–Target Network Analysis

**Figure 3:**
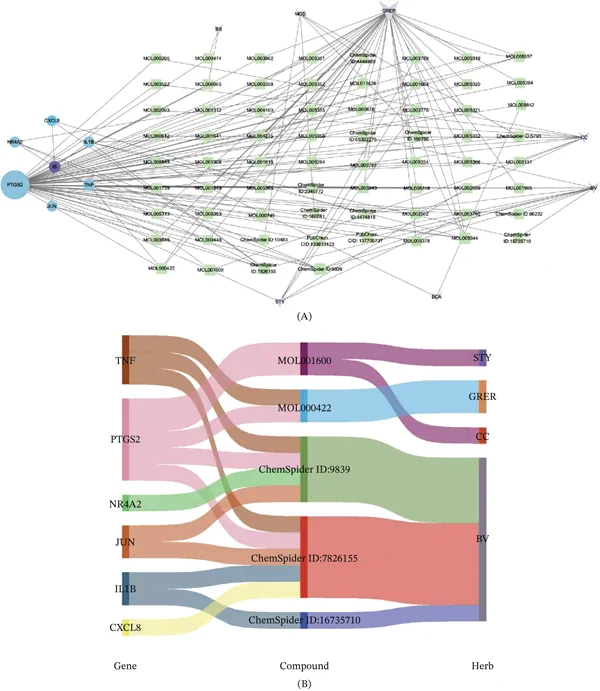
Drug–target–disease network of SBP in the treatment of IS and Sankey diagram of the six core target genes. (A) The diagram shows the connections between herbal sources (triangles), bioactive compounds (squares), target genes (circles), and the disease. Node size indicates degree centrality. (B) Sankey diagram depicting the associations between six core target genes, their linked active compounds, and corresponding herbal origins. The diagram is organized into three panels: the left panel shows the core genes, the middle panel presents their associated bioactive compounds, and the right panel traces these compounds back to their herbal sources. IS: ischemic stroke; GRER: *Ginseng Radix et Rhizoma*; BC: *Bovis Calculus*; CC: *Cinnamomi Cortex*; STY: *Styrax*; BV: *Bufonis Venenum*; MOS: *Moschus*; BS: *Borneolum Syntheticum*.

To preliminarily explore the potential pharmacological association of SBP with IS, a compound–target interaction network was constructed based on the six hub genes. As shown in Figure 3A, the network displays the multicomponent and multitarget characteristics of SBP, wherein multiple active compounds are linked to IS‐related genes. On this basis, five bioactive compounds with relatively high connectivity degrees were further screened, including ChemSpider ID: 9839, ChemSpider ID: 7826155, MOL001600, MOL000422, and ChemSpider ID: 16735710.

A Sankey diagram was generated to visualize the associations among active compounds, six core target genes, and corresponding herbal components (Figure 3B). The diagram illustrated that ChemSpider ID:9839 (bufotenine) and ChemSpider ID:7826155 (bufalin) exhibited relatively higher connectivity, whereas MOL001600 (copaene) and MOL000422 (kaempferol) were each associated with two targets. These observations imply that bufalin and bufotenine may potentially interact with multiple targets simultaneously, whereas other compounds tend to show relatively target‐specific association patterns.

### 3.4. PPI Network Analysis of IS‐Related Genes

**Figure 4:**
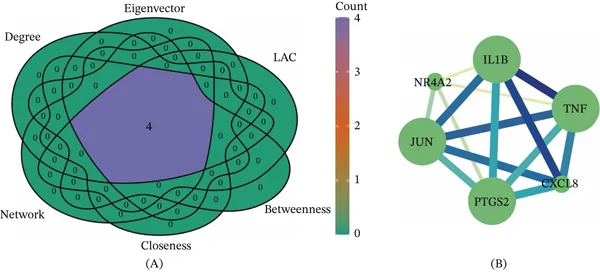
Venn diagram of central targets identified by different algorithms and the core target interaction network. (A) Venn diagram of hub targets identified by different algorithms, with the purple region representing the core targets. (B) The network consists of both core and noncore targets, with the four larger green nodes indicating the main therapeutic targets of SBP for the treatment of IS.

The six intersecting target genes were imported into the STRING database to construct a PPI network, consisting of six nodes and 14 edges. Multiple centrality metrics (degree, betweenness centrality, closeness centrality, eigenvector centrality, local average connectivity, and median network values) were adopted for topological analysis. Based on these network topological features, a subnetwork containing four candidate core targets was screened (Figure 4A). In addition, separate interaction networks were established for the screened core and noncore targets, as illustrated in Figure 4B.

### 3.5. GO and KEGG Enrichment Analyses

**Figure 5:**
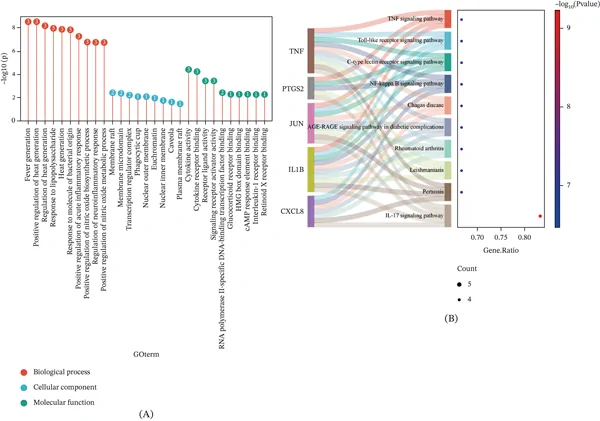
Functional annotation and pathway enrichment analysis of differentially expressed genes. (A) GO function enrichment analysis of potential targets from active ingredients of SBP. (B) KEGG pathways function enrichment analysis of potential targets from active ingredients of SBP.

Figure 5A presents the GO enrichment results of the overlapping targets, with detailed information summarized in Table S4. Enrichment terms were classified into three categories: biological process (BP), cellular component (CC), and molecular function (MF). The horizontal axis shows enriched GO terms, and the vertical axis represents enrichment significance (−log₁₀(P)). In the BP category, terms such as positive regulation of inflammatory response and cytokine‐mediated signaling pathway ranked high in enrichment significance, suggesting a possible correlation of these genes with inflammatory processes. For CC, cytosolic ribosome was prominently enriched, which may reflect the major subcellular distribution characteristics of the related genes. In the MF category, cytokine receptor binding and signaling receptor activator activity showed notable enrichment, implying a potential role in signal transduction.

Figure 5B summarizes the KEGG pathway enrichment results and corresponding regulatory genes (Table S5). Hub genes including TNF, PTGS2, and IL1B were annotated to multiple pro‐inflammatory pathways, such as the TNF, IL‐17, and Toll‐like receptor signaling pathways. The connecting lines represent potential gene–pathway associations, with color intensity corresponding to enrichment significance and node size proportional to the number of enriched genes. The enriched inflammatory pathways showed a general consistency with the results of GO enrichment analysis.

### 3.6. Identification of Core Hub Genes Using Machine Learning Approaches

**Figure 6:**
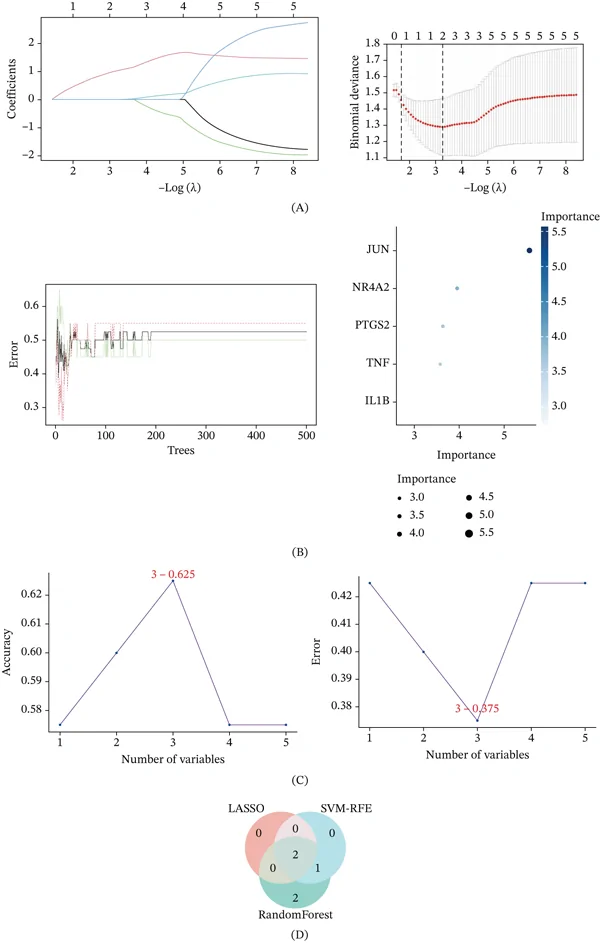
Feature selection and model evaluation for biomarker identification. (A) LASSO regression analysis. (B) Random forest model validation. (C) ROC curve analysis of predictive models. (D) Venn diagram of feature overlap across models.

Figure 6A displays the coefficient trajectories of candidate genes in the LASSO regression analysis. The horizontal axis denotes −log(*λ*), and the vertical axis corresponds to gene coefficient values. As *λ* increased, the coefficients of most genes gradually approached zero, which is consistent with the general characteristic of feature shrinkage in the LASSO algorithm. The right panel depicts the binomial deviance curve against −log(*λ*), where the vertical dashed line marks the optimal *λ* determined by 10‐fold cross‐validation.

Figure 6B presents the results of the RF model. The left panel shows the out‐of‐bag (OOB) error curve, which tended to stabilize around 0.45 with the rise in the number of decision trees. The right panel illustrates the feature importance ranking, with darker color and larger dot size corresponding to higher importance scores. Figure 6C compares the ROC curves of the RF and SVM‐RFE models. Both models yielded AUC values near 0.8, showing acceptable discriminative ability for the studied outcome. Figure 6D provides a Venn diagram of candidate features screened by LASSO, SVM‐RFE, and RF algorithms. Two overlapping features were shared across the three algorithms, which may imply a potential consistent relevance to IS.

### 3.7. Validation and Diagnostic Performance of the Selected Genes

**Figure 7:**
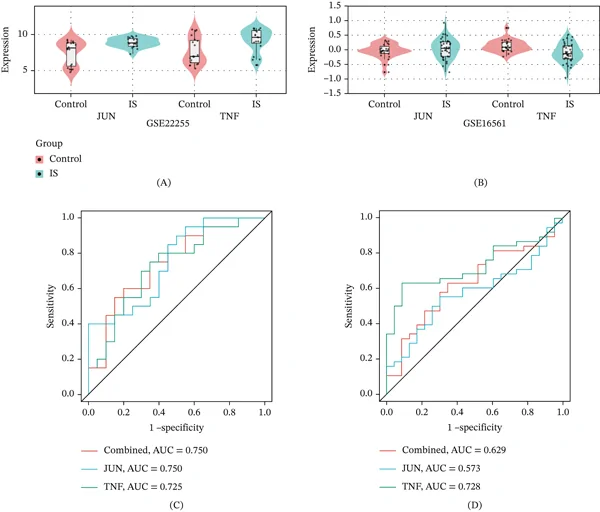
Validation of candidate genes and evaluation of their diagnostic efficacy. (A) Expression levels of JUN and TNF show significant differences in the GSE22255 dataset (*p* < 0.05). (B) Expression levels of JUN and TNF show significant differences in the GSE16561 dataset (*p* < 0.05). (C) ROC curve analysis for the GSE22255 dataset. (D) ROC curve analysis for the GSE16561 dataset, supporting the combined diagnostic efficacy of the two genes.

Violin and box plots of the training (Figure 7A) and validation (Figure 7B) cohorts showed observable expression differences between the IS and control groups, with overall expression patterns being comparable across the two datasets. ROC curve analysis was further performed to evaluate the potential diagnostic implication of the screened genes. In the training cohort (Figure 7C), JUN (AUC = 0.750), TNF (AUC = 0.725), and the combined gene panel (AUC = 0.750) exhibited moderate discriminatory capacity. In the validation cohort (Figure 7D), TNF maintained a comparable AUC of 0.728, whereas the discriminatory performance of JUN declined to 0.573. The combined gene panel yielded an AUC of 0.698, without presenting evident improvement compared with TNF alone. Collectively, these results suggest that TNF may exhibit relatively stable expression performance across cohorts, whereas JUN shows limited additional contribution to discriminatory capability.

### 3.8. GSEA of the Two Key Targets

**Figure 8:**
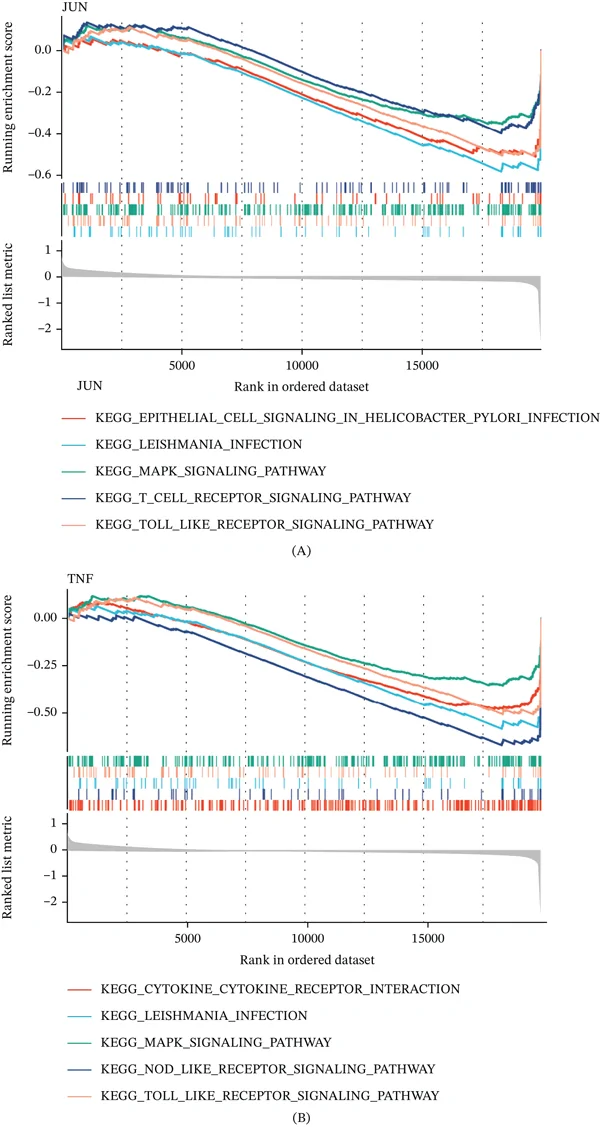
Gene set enrichment analysis of key target genes. (A) KEGG enrichment analysis of JUN. (B) KEGG enrichment analysis of TNF.

As shown in Figure 8A,B, multiple inflammation‐related pathways exhibited significant enrichment, which may reflect a possible link between innate immune dysregulation and IS progression (Tables S6–S7). Representative pathways, including the Toll‐like receptor, NOD‐like receptor, and MAPK signaling pathways, were prominently enriched, potentially suggesting that danger signals induced by ischemic injury might correlate with inflammatory responses, subsequent cytokine release, and immune cell infiltration. Furthermore, enrichment of the cytokine–cytokine receptor interaction pathway implies a putative complex immune regulatory network underlying cerebral ischemia. Enrichment of the T cell receptor signaling and leishmaniasis pathways may also hint at a possible association of T cell activation and macrophage‐related inflammation with the postischemic immune microenvironment. Overall, these enrichment results point to a broad potential relevance of immune and inflammatory processes in the progression of IS.

### 3.9. Immune Cell Infiltration Analysis

**Figure 9:**
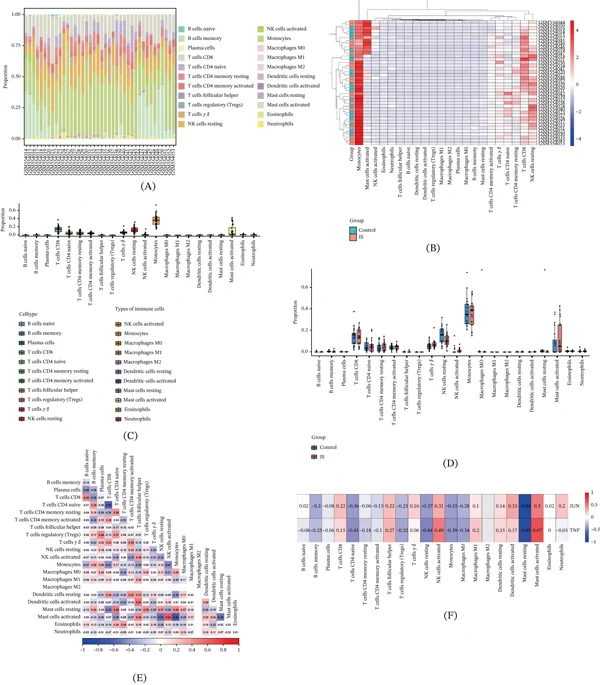
Comprehensive characterization of immune cell infiltration patterns and their molecular associations. (A) Immune cell composition across samples. (B) Clustering of immune cell fractions. (C) Distribution of representative immune cell types. (D) Immune cell differences between control and IS groups. (E) Correlation matrix of immune cell types. (F) Correlations between key genes and immune cells.

Immune cell infiltration analysis was conducted on the training dataset (GSE22255) using the CIBERSORT algorithm to estimate the relative proportions of 22 immune cell subsets (Figure 9A, Table S8). Sixteen immune cell subsets presented statistically different infiltration levels between IS and control samples (*p* < 0.05). Among them, M1/M0 macrophages, follicular helper T cells, regulatory T cells (Tregs), and activated dendritic cells tended to show higher proportions in IS samples (Figure 9B), which may suggest altered infiltration patterns of innate immune cells. Further analyses (Figure 9C–E) indicated that IS samples tended to be dominated by innate immune cells, accompanied by a relative reduction in adaptive immune cell proportions. Hierarchical clustering analysis (Figure 9D) showed an IS subgroup with enriched myeloid cells and reduced lymphocyte abundance. Correlation analysis (Figure 9E–F) showed positive correlations among innate immune cell subsets, negative correlations between innate and adaptive immune cells, as well as correlative relationships between hub genes and specific immune cell types.

Overall, these analyses depict distinct immune cell patterns in IS, including differential infiltration, compositional distribution changes, intercellular correlation, and gene–immune cell associations, which may imply a potential link between immune dysregulation and IS progression.

### 3.10. Molecular Docking Validation

**Figure 10:**
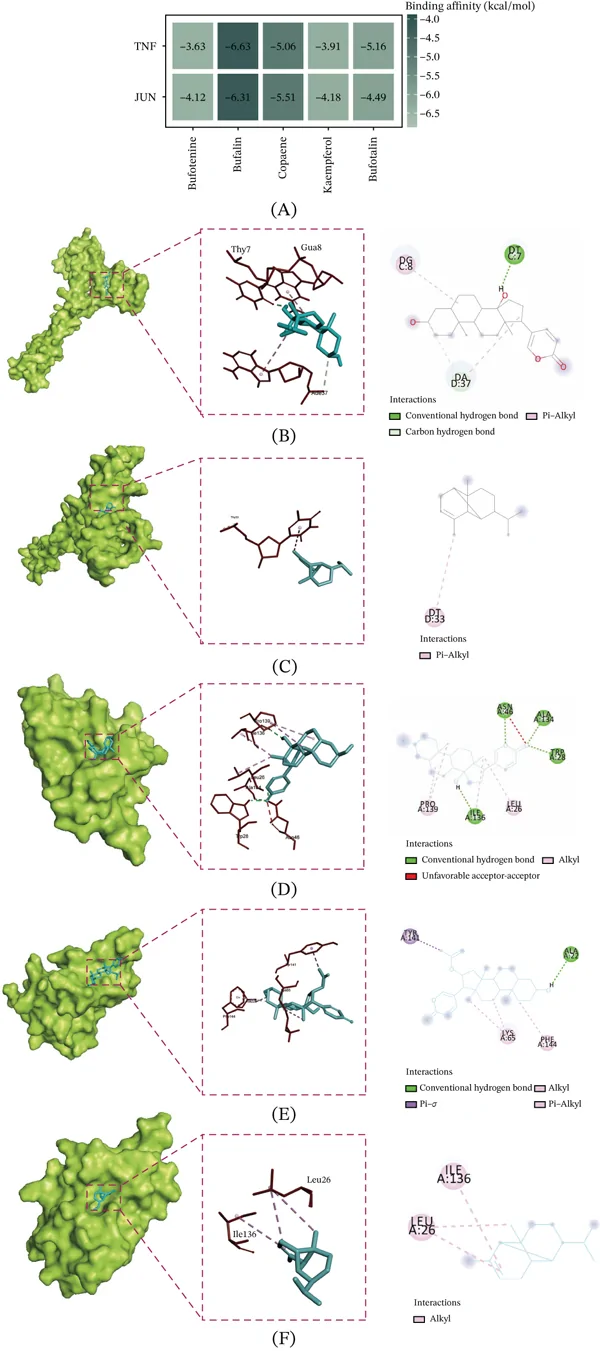
Prediction of drug–target interactions for candidate genes. (A) The heat map visualizes the binding affinity scores obtained from molecular docking simulations between two representative target proteins and their corresponding active compounds. (B–F) Schematic diagrams of representative molecular docking pairs: (B) JUN–bufalin, (C) JUN–copaene, (D) TNF–bufalin, (E) TNF–bufotalin, and (F) TNF–copaene.

Binding mode analysis was conducted to explore the predicted interaction patterns between SBP compounds and core targets (Figure 10B–F). The in silico results suggest that these compounds may occupy the binding pockets of TNF and JUN through potential noncovalent interactions, including hydrogen bonds, hydrophobic contacts, and *π*–*π* stacking with key amino acid residues. Hydrophobic residues such as Ile, Leu, and Phe may contribute to the stability of the predicted binding, whereas polar residues might form directional hydrogen bonds that could help maintain ligand orientation.

Among all tested compounds, bufalin exhibited the relatively strongest predicted binding affinities for both targets: −6.63 kcal/mol for TNF and −6.31 kcal/mol for JUN, with both values falling within the range of predicted moderate binding. The predicted binding affinity for TNF was comparable to that of the known inhibitor SPD‐304 (reported range: −5 to −8 kcal/mol) [33], whereas the predicted affinity for JUN was consistent with previously reported in silico results for inhibitors such as SP600125 [34]. All other candidate compounds also showed moderate negative predicted binding energies, which may indicate thermodynamically favorable interactions in the computational model.

Overall, these molecular docking results imply a potential capacity of SBP components to bind TNF and JUN in silico, which might be associated with the modulation of inflammatory signaling pathways. These computational findings are preliminary and require further validation through in vitro and in vivo experimental studies.

### 3.11. MD Simulation

MD simulations were performed to predict the conformational stability and binding energetics of TNF– and JUN–ligand complexes. As shown in Figure 11A, RMSD analysis indicated that TNF–ligand complexes tended to maintain relatively stable backbone conformations, with RMSD values ranging from 0.1 to 0.2 nm. In contrast, JUN–ligand complexes showed relatively larger fluctuations, with RMSD values around 0.35 nm, which may reflect a potential inherent flexibility of the bZIP DNA‐binding domain. Nevertheless, all JUN complexes appeared to retain structural integrity without obvious ligand dissociation in the simulated system.

Figure 11Molecular dynamics simulation of drug–target complexes. (A) Root mean square displacement, (B) root mean square fluctuation, (C) radius of gyration, (D) solvent accessible surface area, (E) hydrogen bonds, and (F–J) free energy landscape (FEL).

**Figure:**
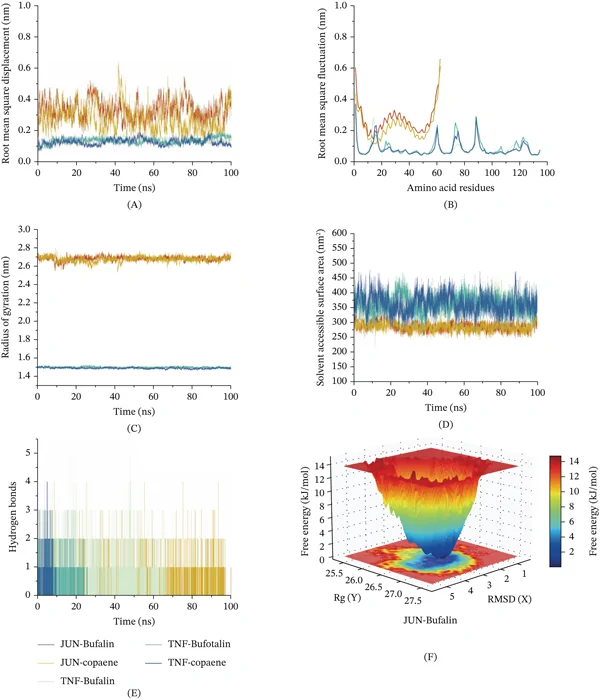
(a)

**Figure:**
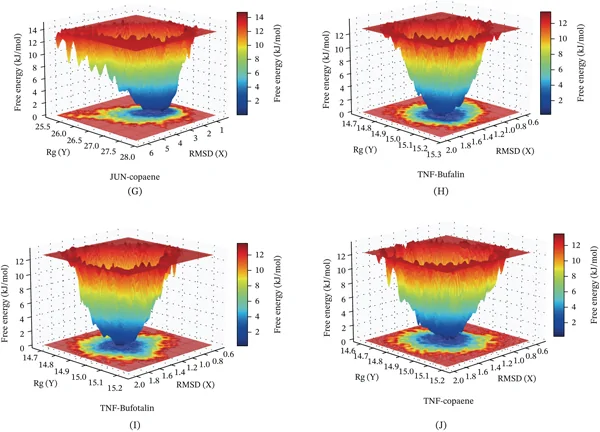
(b)

**Figure 12:**
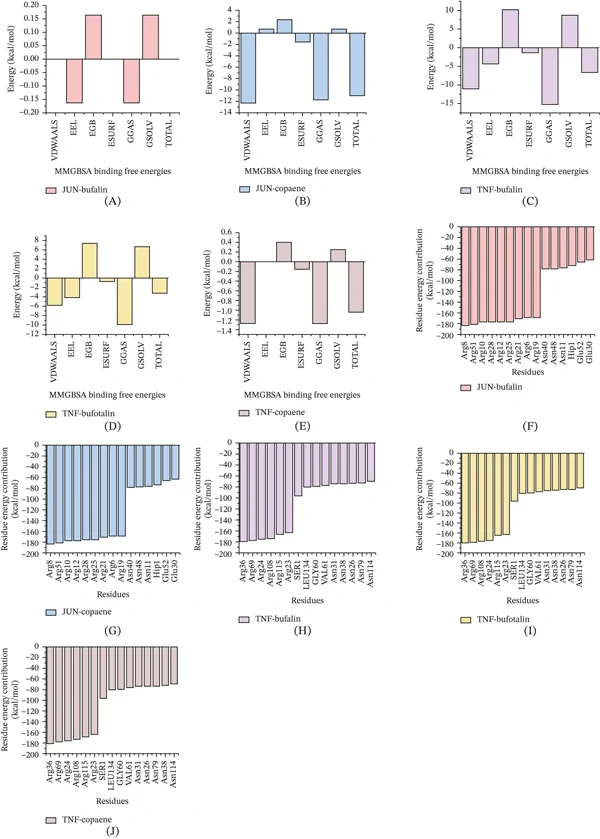
MM/GBSA binding free energy decomposition: energetic profiles of protein–ligand complexes. (A–E) Component contributions to MM/GBSA binding free energies. (F–J) Residue‐level contributions to binding free energy.

RMSF analysis (Figure 11B) showed restricted residue motion in TNF complexes (RMSF < 0.4 nm), whereas JUN complexes exhibited higher flexibility, particularly within the predicted binding regions. Subsequent Rg and SASA analyses (Figure 11C,D) were consistent with a relatively compact and stable global conformation of TNF complexes, whereas JUN complexes showed larger structural dimensions and greater flexibility, which may align with the distinct structural characteristics of the two proteins. Hydrogen bond analysis (Figure 11E) suggested the presence of relatively stable hydrogen bond interactions (1–3 bonds) in TNF complexes, whereas JUN complexes displayed more variable hydrogen bond formation (0–4 bonds) during the simulation period. Free energy landscape (FEL) analysis (Figure 11F–J) also indicated more concentrated low‐energy conformations for TNF complexes and more dispersed energy states for JUN complexes in the simulated system. MM/GBSA binding energy calculations predicted that the TNF–bufalin complex had the relatively most favorable binding energy, with key residues (Ala80 and Asp86) potentially contributing to the stability of the predicted binding. JUN complexes showed distinct predicted residue interaction patterns (Figure 12C,H).

Overall, these MD simulation results imply potential target‐specific differences in conformation and energetics among the analyzed complexes, with the TNF–bufalin complex exhibiting relatively higher stability and binding affinity in the computational simulations.

## 4. Discussion

IS remains a leading global cause of mortality and long‐term disability, yet effective pharmacotherapies that directly target the complex molecular cascades of ischemic injury are still limited. In this context, the multifactorial nature of IS—characterized by the interplay of neuroinflammation, oxidative stress, apoptosis, and vascular dysfunction—poses a substantial challenge to conventional single‐target therapeutic approaches and may underscore the need for integrative analytical strategies. Increasing evidence suggests that IS may represent a systemic process beyond a localized brain injury, involving a complex and potentially bidirectional brain–heart axis associated with inflammation and autonomic dysregulation. Despite growing interest in this area, the molecular mechanisms underlying stroke–heart crosstalk remain incompletely understood, which may limit effective clinical translation. From this broader perspective, cardiovascular agents might offer additional therapeutic potential in IS, although this possibility deserves further systematic investigation [35]. In this study, we employed a systems‐level computational framework integrating systems pharmacology, transcriptomic analysis, and machine learning–based feature selection to explore the potential mechanisms associated with the effects of SBP in IS.

In this study, we applied an integrative computational framework combining systems pharmacology, transcriptomic analysis, and machine learning‐based feature selection to explore the predicted molecular interactions between SBP constituents and IS‐related pathological processes. We constructed a compound–target network involving 126 putative bioactive compounds and 796 candidate targets. By integrating disease‐related genes and transcriptomic datasets, we further prioritized genes potentially closely associated with ischemic pathology. Functional enrichment analysis suggested that these targets were notably enriched in inflammatory and immune regulatory pathways, which is consistent with the well‐recognized potential role of neuroinflammation and immune dysregulation in ischemic brain injury.

PPI analysis and multiple machine learning algorithms consistently identified six candidate hub genes: CXCL8, IL1B, JUN, NR4A2, PTGS2, and TNF. These genes may be potentially involved in inflammatory signaling and cellular stress responses during ischemic injury [36]. For instance, TNF has been reported to function as a pivotal inflammatory mediator that contributes to endothelial dysfunction, ischemia–reperfusion injury, and blood–brain barrier disruption in MCAO models. Inhibition of TNF signaling has been shown to reduce neuronal apoptosis and improve neurological outcomes in preclinical studies [37]. JUN, a key component of the AP‐1 transcription complex, is known to regulate stress‐responsive gene expression, apoptosis, and inflammatory signaling. Excessive JUN activation has been associated with exacerbated neuronal injury in both in vitro and in vivo ischemia models [38]. Similarly, CXCL8 and IL1B are pro‐inflammatory cytokines that promote the recruitment and activation of macrophages and neutrophils, thereby potentially amplifying neuroinflammation [39]. PTGS2 participates in prostaglandin biosynthesis and may contribute to inflammatory responses and vascular dysfunction during cerebral ischemia [40]. NR4A2, a neuroprotective nuclear receptor, is also implicated in neuronal survival under ischemic stress [41]. Collectively, these candidate hub genes may be closely linked to inflammatory and stress‐related pathways potentially central to IS pathogenesis. Notably, the high mortality and long‐term disability rates of IS, which constitute a heavy global disease burden, are reported to be closely associated with the inflammatory responses mediated by genes such as TNF and JUN. These genes have been widely reported to participate in inflammatory signaling and cellular stress responses associated with ischemic injury.

Consistent with this pathological background, JUN and TNF were relatively stably prioritized across multiple machine learning models and validation procedures, suggesting a potential robust association with IS in the computational framework. Immune infiltration analysis further indicated increased proportions of macrophages, activated dendritic cells, and certain T cell subsets in IS samples. The observed correlations between JUN/TNF expression and immune cell infiltration imply that these candidate hub genes may potentially modulate immune regulatory processes during postischemic inflammation.

To enhance the reliability of the in silico results, we adopted multiple analytical strategies. Screening thresholds were selected based on common standards and dataset properties. Sensitivity analysis suggested consistent gene prioritization under alternative parameters, which may support the stability of the predicted results. To mitigate cohort‐specific biases, we employed two independent transcriptome datasets for model training and external validation. For example, the training dataset GSE22255 included 20 IS patients, whereas the independent validation dataset GSE16561 included 39 IS patients, representing different sample sizes, demographic characteristics, and experimental platforms. These differences may lead to variations in gene expression profiles and statistical outcomes, thereby quantitatively affecting the ranking and prioritization of candidate hub genes.

Furthermore, different compound–target databases (TCMSP, HERB, SwissTargetPrediction) use distinct inclusion criteria and experimental evidence levels, leading to quantitative differences in the number and validity of predicted targets. For instance, the same compound may obtain dozens of predicted targets from structure‐based databases such as SwissTargetPrediction, but only a small number of experimentally verified targets from TCMSP, which may change the overall target pool size, skew PPI network topology, and further influence the ranking and priority of candidate hub genes including TNF and JUN. To address this, we standardized annotations using PubChem CID and HUGO gene symbols, removed duplicate records, and retained only disease‐relevant candidate targets. Machine learning models were evaluated using stratified cross‐validation on the training set and independently validated on GSE16561 to minimize overfitting. The high AUC values in both sets suggest the potential discriminative capacity of the identified gene signature in the computational analysis.

We refined the comparative analysis of computational approaches to investigate their trade‐offs between data availability, interpretability, and predictive performance under clinically relevant scenarios. Two independent transcriptomic datasets from the GEO database were used: the training set GSE22255 included 40 samples (20 healthy controls and 20 IS patients), whereas the validation set GSE16561 included 63 samples (24 healthy controls and 39 IS patients). These datasets differed in sample size, demographic composition, and experimental platforms, providing a realistic heterogeneous setting for evaluating method robustness in silico. Traditional PPI network–based methods offer superior interpretability, as candidate hub genes can be linked to network topology and known biological functions. However, they depend highly on the completeness of existing interaction data, which may restrict their stability and predictive performance in small or heterogeneous datasets such as GSE22255. By comparison, machine learning–based models (RF, SVM‐RFE, and LASSO) show stronger tolerance to data heterogeneity and better predictive performance in the current analysis, supported by the high AUC values in both training and validation. Nevertheless, machine learning approaches typically have lower interpretability and may be more prone to overfitting under limited sample sizes. On the basis of these characteristics, practical selection criteria can be summarized: When sample size is limited and biological interpretability is prioritized, PPI network analysis may be more reliable and transparent; when dataset heterogeneity is high and predictive performance is emphasized, machine learning models may be more advantageous owing to their better adaptability. By integrating PPI network analysis, multiple machine learning strategies, and multidatabase integration, we aimed to balance interpretability, robustness to heterogeneity, and predictive performance in the computational framework. The use of GSE22255 for training and GSE16561 for external validation further suggests the potential stability and generalizability of our candidate hub gene prioritization pipeline. Notably, this study was not designed to identify novel IS‐related genes, as most identified candidate hub genes have well‐documented roles in inflammatory signaling. Instead, we aimed to systematically link these established pathological nodes to the multicomponent pharmacological network of SBP using an integrated bioinformatic framework.

Consistent with our integrated computational strategy and in consideration of database‐related variability discussed above, among the predicted active compounds, bufalin and bufotalin, two representative bufadienolides from *Bufonis Venenum*, have been reported to exert anti‐inflammatory effects. These compounds can suppress TLR4/NF‐*κ*B/NLRP3 signaling and reduce the production of pro‐inflammatory cytokines including TNF‐*α* and IL‐1*β*, which are reported to be key drivers of neuroinflammation in cerebral ischemia [42, 43]. The Nrf2/HO‐1 antioxidant pathway also contributes to ischemic neuroprotection in preclinical studies [44, 45]. Although not directly validated in the current study, our predicted compound–target interactions show consistency with experimentally established mechanisms reported in the literature. Notably, most supporting evidence originates from preclinical animal models, which may not fully recapitulate the complexity, comorbidities, and heterogeneity of human IS, representing a well‐recognized challenge in translational research. This concordance nevertheless suggests the potential reliability of our network‐based approach, while also emphasizing the need to account for data heterogeneity and model differences when translating in silico findings toward clinical applications.

Overall, this study provides a systems‐level computational framework for connecting the multicomponent network of SBP with potential pathological pathways in IS, offering potential novel insights into the putative neuroprotective mechanisms of SBP from a holistic perspective. This integrative approach, combining computational prediction and multi‐omics analysis, prioritizes key candidate hub genes and predicted compound–target interactions that may serve as potential targets for further mechanistic exploration. While the findings provide a preliminary foundation for understanding SBP′s potential role in IS treatment, the translational potential of these computational hypotheses remains to be confirmed through experimental studies. As such, subsequent research should focus on validating the proposed mechanisms through rigorous experimental and clinical studies, which will be critical for translating the current theoretical framework into tangible clinical applications.

## 5. Limitations

Several limitations of this study should be acknowledged. First, all analyses were based on public transcriptomic and pharmacological databases, which may introduce bias. Patient cohorts differed in sample size, age, and comorbidities (e.g., GSE22255 vs. GSE16561), and experimental platforms varied in probe design and normalization methods. In addition, compound–target databases differed in coverage and evidence levels, which may influence target prediction and hub gene identification related to IS and cardiovascular regulatory mechanisms. Second, all compound–target predictions, molecular docking, and simulation results represent in silico predictions only, without direct experimental validation. These findings should therefore be regarded as hypothesis‐generating rather than conclusive evidence for the mechanisms of SBP in IS. Third, despite independent external validation, inherent heterogeneity across transcriptomic datasets may still affect the reproducibility of gene signatures and predictive models.

Finally, this study lacks experimental verification using in vitro assays or in vivo MCAO models to confirm the regulatory effects of SBP on the key hub genes and inflammatory pathways identified in this study.

## 6. Conclusion

This study applied an integrative computational strategy combining systems pharmacology, bioinformatics, and machine learning to investigate the potential mechanisms of SBP in IS. Our results indicate that SBP‐associated targets are predominantly enriched in inflammatory and immune‐related pathways, with JUN and TNF identified as key molecular nodes linking its cardiovascular actions to neuroprotection in IS. These findings provide computational insights into the multitarget pharmacological effects of SBP. However, considering that the present results are derived from in silico analyses, further experimental and clinical studies are warranted to validate the biological relevance and therapeutic potential of these observations.

## Author Contributions

**Jingju Fu:** writing – original draft, writing – review and editing, validation, data curation, conceptualization, funding acquisition. **Yukun Wang:** writing – original draft, methodology, data curation. **Xinyi Li:** writing – review and editing, software, funding acquisition. **Xue Dong:** writing – review and editing, conceptualization, project administration, methodology.

## Funding

This work was supported by the Shandong Provincial Natural Science Foundation [No. ZR2025QC1379, No. ZR2022LZY015] and the Shandong Province Traditional Chinese Medicine Science and Technology Project [No. Q‐2023084, No. Q‐2023021, No. M‐20242211, No. M‐2023088].

## Conflicts of Interest

The authors declare no conflicts of interest.

## Data Availability

The data that support the findings of this study are available on request from the corresponding author.
